# Pre-Hospital Lactatemia Predicts 30-Day Mortality in Patients with Septic Shock—Preliminary Results from the LAPHSUS Study

**DOI:** 10.3390/jcm9103290

**Published:** 2020-10-14

**Authors:** Romain Jouffroy, Teddy Léguillier, Basile Gilbert, Jean Pierre Tourtier, Emmanuel Bloch-Laine, Patrick Ecollan, Vincent Bounes, Josiane Boularan, Papa Gueye-Ngalgou, Valérie Nivet-Antoine, Jean-Louis Beaudeux, Benoit Vivien

**Affiliations:** 1 Intensive Care Unit, Ambroise Paré Hospital—Assistance Publique Hôpitaux de Paris, 92100 Boulogne Billancourt, France; 2 Intensive Care Unit, Anaesthesiology, SAMU, Necker Enfants Malades Hospital, Assistance Publique—Hôpitaux de Paris, 75015 Paris, France; benoit.vivien@aphp.fr; 3 Emergency Medicine Department, 1 Place Jules Renard, Paris Fire Brigade, 75017 Paris, France; jean-pierre.tourtier@intradef.gouv.fr; 4 Department of Clinical Biochemistry, Necker Hospital, Assistance Publique—Hôpitaux de Paris, 75015 Paris, France; teddy.leguillier@aphp.fr (T.L.); valerie.nivet-antoine@parisdescartes.fr (V.N.-A.); jean-louis.beaudeux@parisdescartes.fr (J.-L.B.); 5 UMR-S1140, Faculty of Pharmaceutical Sciences of Paris, Paris Descartes University, 75015 Paris, France; 6 Department of Emergency Medicine, SAMU 31, University Hospital of Toulouse, 31000 Toulouse, France; basilegilbert2@gmail.com (B.G.); bounes.v@chu-toulouse.fr (V.B.); 7 Emergency Department, Cochin Hospital, Paris, France & Emergency Department, SMUR, Hôtel Dieu Hospital, 75014 Paris, France; emmanuel.blochlaine@aphp.fr; 8 Intensive Care Unit, SMUR, Pitie Salpêtriere Hospital, 47 Boulevard de l’Hôpital, 75013 Paris, France; patrick.ecollan@aphp.fr; 9 Emergency Department, SAMU 31, Castres Hospital, 81108 Castres, France; josiane.boularan@chic-cm.fr; 10 Emergency Department, SAMU 972, CHU de Martinique Pierre Zobda—Quitman Hospital, 90632 Fort-de-France Martinique, France; Papa.GUEYE@chu-martinique.fr

**Keywords:** severe sepsis, septic shock, blood lactate, pre-hospital setting, prediction

## Abstract

Background: Assessment of disease severity in patients with septic shock (SS) is crucial in determining optimal level of care. In both pre- and in-hospital settings, the clinical picture alone is not sufficient for assessing disease severity and outcomes. Because blood lactate level is included in the clinical criteria of SS it should be considered to improve the assessment of its severity. This study aims to investigate the relationship between pre-hospital blood lactate level and 30-day mortality in patients with SS. Methods: From 15 April 2017 to 15 April 2019, patients with SS requiring pre-hospital Mobile Intensive Care Unit intervention (MICU) were prospectively included in the LAPHSUS study, an observational, non-randomized controlled study. Pre-hospital blood lactate levels were measured at the time of first contact between the patients and the MICU. Results: Among the 183 patients with septic shock requiring action by the MICU drawn at random from LAPHSUS study patients, six (3%) were lost to follow-up on the 30th day and thus 177 (97%) were analyzed for blood lactate levels (mean age 70 ± 14 years). Pulmonary, urinary and digestive infections were probably the cause of the SS in respectively 58%, 21% and 11% of the cases. The 30-day overall mortality was 32%. Mean pre-hospital lactatemia was significantly different between patients who died and those who survived (respectively 7.1 ± 4.0 mmol/L vs. 5.9 ± 3.5 mmol/L, *p* < 10^−3^). Using Cox regression analysis adjusted for potential confounders we showed that a pre-hospital blood lactate level ≥ 4 mmol/L significantly predicted 30-day mortality in patients with SS (adjusted hazard ratio = 2.37, 95%CI (1.01–5.57), *p* = 0.04). Conclusion: In this study, we showed that pre-hospital lactatemia predicts 30-day mortality in patients with septic shock handled by the MICU. Further studies will be needed to evaluate if pre-hospital lactatemia alone or combined with clinical scores could affect the triage decision-making process for those patients.

## 1. Introduction

Every year, sepsis affects more than 30 million people worldwide [1,2,3], leading to 11 million deaths. This represents around 20% of all global deaths [3] and one-third to one-half of all in-hospital deaths [4]. Despite recent advances in the treatment of sepsis and SS (septic shock), the mortality rate in patients admitted to the intensive care unit (ICU) remains stable, ranging from 10 to 20% for sepsis and from 50 to 60% for SS [5,6,7]. In 2016, the “sepsis 3” conference and the Center for Disease Control and Prevention emphasized that early recognition, severity assessment and early treatment were priorities in order to improve the survival of patients with sepsis [8].

Because most sepsis (70%) occurs in a pre-hospital environment [9], a better assessment of those patients by the MICU associated with an optimized orientation between the ED and the ICU seems the best way to improve the survival rate of those patients. In France, evaluation of sepsis severity is based on medical history, clinical signs and laboratory-measured biomarkers. In a hospital setting, this evaluation is used to manage patient orientation between ED and ICU, however, in a pre-hospital setting, the decision-making is entirely based on clinical signs according to the French SFAR-SRLF conference 2005 [10]. 

Tissue perfusion may be assessed either clinically (blood pressure, pulse, capillary refill time and/or skin mottling score) or biologically [11] at hospital level. Among patients with sepsis, a subset progress to septic shock in which profound macro/microcirculatory and cellular metabolism abnormalities are associated with increased blood lactate levels. Previous studies reported that the mortality rate was better correlated with blood lactate levels higher than 4 mmol/L than with refractory hypotension after vascular filling in patients with SS (systolic blood pressure < 90 mmHg after 1 L of fluid expansion) [12,13]. Furthermore, it was shown that increased blood lactate level is a better prognostic indicator than other organ dysfunction biomarkers to assess sepsis severity [14,15,16,17]. Finally, blood lactate is the only biomarker of tissue perfusion validated in an extra hospital setting [18].

For the first time, this study aims to investigate the relationship between pre-hospital blood lactate level and 30-day mortality in patients with SS.

## 2. Methods

### 2.1. Patients

Emergency medical services in France are represented by a public health control organization, the Urgent Medical Aid Service (SAMU), which provides medical response to emergency situations. SAMU also has a mobile intensive care unit, the Mobile Emergency and Resuscitation Service (SMUR), which provides out-of-hospital treatment and transport to definitive care. Briefly, the central component of SAMU is the dispatch center where a team of physicians and assistants answer calls, triage the patients’ complaints and respond to them [19]. In the case of life-threatening emergencies, a mobile intensive care unit (MICU), composed of a driver, a nurse and an emergency physician, is dispatched to the scene. MICU is equipped with medical devices and drugs allowing initial management of the main organ deficiencies (neurological, respiratory and cardiovascular) [20]. From 15 April 2017 to 15 April 2019, patients (age ≥ 18 years) with a diagnosis of septic shock according to the SFAR-SRLF criteria [10] were prospectively included in this study by the MICU physicians of 9 hospital centers (Necker-Enfants malades Hospital; Lariboisière Hospital; La Pitié Salpêtrière Hospital; Hotel Dieu Hospital; APHP, Paris, France; The Paris Fire Brigade Paris, France; The Toulouse University Health Center, Toulouse, France; and the Castres Hospital, Castres, France). Patients younger than 18 years, and/or pregnant, and/or with serious comorbid conditions with an unknown pre-hospital life support and/or with guardianship or curatorship were not included in this study. Blood lactate levels were measured at the time of first contact between the patients and the MICU using a point of care medical device (StatStrip^®^ Lactates, Nova Biomedical, Waltham, MA, USA). A previous study has demonstrated the comparability and transferability between this medical device and the central laboratory analyzers [18]. A sequential organ failure assessment (SOFA) score [21] and a simplified acute physiology score (SAPS 2) [22] were calculated 24 h after ICU admission. This is a preliminary analysis among a sample of 183 randomly selected LAPHSUS patients.

### 2.2. Ethical Considerations

This prospective, observational, non-randomized controlled study was performed according to international guidelines (Declaration of Helsinki, International Conference on Harmonization and the WHO Good Clinical Practice standards, including certification by an external audit) after obtaining written informed consent of all participants. The study was approved by an ethics committee and institutional review boards (CPP 2015-08-03 SC for the local ethics committee and ID RCB number: 2015-A01068-41 for the National Heart Agency) and registered in the ClinicalTrials.gov database (NCT03831685) as previously published [23].

### 2.3. Statistical Analysis

Results are expressed as the mean with standard deviation for quantitative parameters with a normal distribution, as median with interquartile range (Q1–Q3) for parameters with a non-gaussian distribution and as absolute value and percentage for qualitative parameters. The primary outcome was the 30-day mortality rate and the secondary outcomes were the in-ICU and in-hospital length of stay. Univariate and multivariate analyses were first performed to evaluate the relationship between each covariate and 30-day mortality. Pre-hospital lactatemia level was analyzed as a continuous and as a binary variable using a threshold of lactate ≥4 mmol/L according to previous studies [8,14,15,16,17,24,25,26,27,28,29,30,31,32,33,34,35,36]. Second, the relationship between 30-day mortality and pre-hospital lactatemia was assessed using logistic regression including potential confounders. Third, to reduce the effect of confounders on 30-day mortality and on pre-hospital lactatemia, a propensity score matching was used to balance the differences in baseline characteristics between patients with pre-hospital lactatemia ≥ 4 mmol/L and those with pre-hospital lactatemia < 4 mmol/L. The propensity score, i.e., the probability of pre-hospital lactatemia ≥ 4 mmol/L, was estimated using logistic regression based on potential confounders: age and in-hospital length of stay for 30-day mortality and initial mean arterial blood pressure and initial pulse oximetry for initial blood lactate level [37]. Genetic matching method was used to match patients based on the logit of the propensity score [38]. The balance of covariates after matching was assessed by absolute mean differences with a considered acceptable threshold of 10% [39]. Fourth, a survival analysis using Cox proportional hazards regression was used to compare 30-day mortality of patients with and without pre-hospital lactatemia ≥ 4 mmol/L in the propensity score-matched cohort. Proportional hazards assumption was verified for each Cox model variable by Kaplan–Meier curve and log-rank test. Results are expressed by adjusted hazard ratio (HRa) with 95% confidence interval (95%CI). All tests were 2-sided with a statistically significant *p*-value < 0.05. All analyses were performed using R 3.4.2 (http://www.R-project.org; the R Foundation for Statistical Computing, Vienna, Austria).

The dataset supporting the conclusions of this article is included within the article and its additional file.

## 3. Results

### 3.1. Patient Characteristics

**Figure 1:**
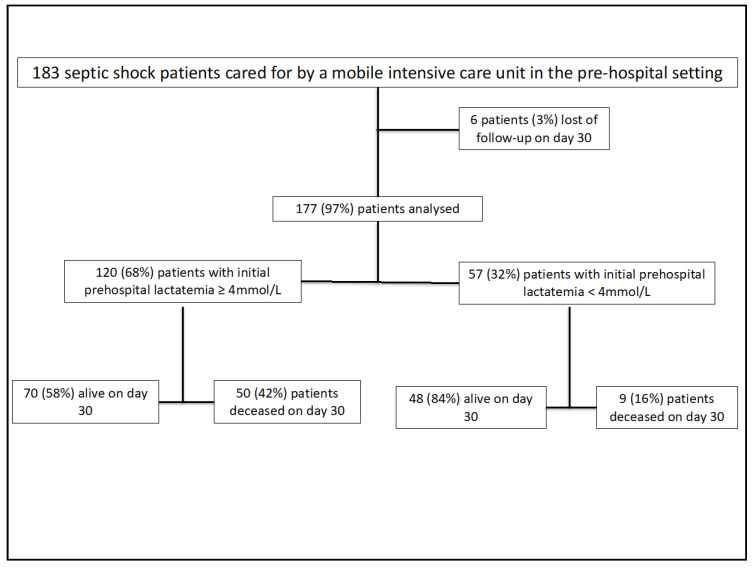
Flow chart.

A total of 183 patients with septic shock requiring action by the MICU were drawn at random among LAPHSUS study patients. Among them, 6 (3%) were lost to follow-up on the 30th day thus 177 (97%) were analyzed (Figure 1).

The SS group was composed of 124 males and 53 females with a mean age of 70 ± 14 years. Among patients with SS, 34 (19%) had at least two comorbidities (Table 1).

The average ICU length of stay was 6 (3–10) days and the average length of stay in a hospital was 14 (8–22) days. Pulmonary, urinary and digestive infections were probably the cause of the SS in, respectively, 58%, 21% and 11% of the cases (Table 2).

The 30-day overall mortality reached 32%. No significant difference in the duration of pre-hospital stage was observed between patients who survived and those who died (83 ± 29 min vs. 83 ± 29 min respectively, *p* = 0.97; Table 1). All patients with SS received crystalloids and no significant difference in pre-hospital fluid expansion was found between living and deceased patients (1067 ± 592 mL vs. 980 ± 575 mL respectively, *p* = 0.36; Table 1). Among patients who received norepinephrine (36%), no significant dose or drug-related difference was found between the groups (1.0 (0.5–2.0) vs. 1.0 (0.7–2.0) mg.h^−1^, respectively, *p* = 0.67; Table 1). Finally, no significant difference in terms of survival was observed between patients who received or did not receive antibiotics (30% died in both groups, *p* = 0.89; Table 1). 

Among the 54 patients (37%) who received antibiotics prior to hospital admission 75% were treated with 3rd generation cephalosporin, 52% with cefotaxime and 22% with ceftriaxone.

### 3.2. Main Measurement

Blood lactate measurements (BLM) from 177 patients with SS were collected in this observational study. The mean pre-hospital lactatemia in the study population was 6.3 ± 3.7 mmol/L and differed significantly between living and deceased patients (5.9 ± 3.5 mmol/L vs. 7.1 ± 4.0 mmol/L, respectively, *p* < 10^−3^). Pre-hospital hyperlactatemia ≥4 mmol/L was associated with a higher 30-day mortality than in patients with lactate < 4 mmol/L (40% vs. 16%; *p* < 0.001). Table 3 depicts the comparison between patient characteristics among those with initial pre-hospital lactatemia < 4 mmol/L and those with initial pre-hospital lactatemia ≥ 4 mmol/L.

In univariate analysis, 30-day mortality was significantly associated with pre-hospital blood lactate level (*p* < 0.001), SOFA score (*p* < 0.001), SAPS2 score (*p* < 0.001), age (*p* = 0.009), glycemia (*p* = 0.03) and in-hospital length of stay (*p* < 0.001) (Table 1). Considering pre-hospital lactatemia as a continuous variable, we observed a significant association with lactatemia and 30-day mortality (OR (95%CI) = 1.09 (1.01–1.20), *p* = 0.04), for in-ICU length of stay (OR (95%CI) = 1.99 (1.26–3.18)), *p* = 0.04 but not for in-hospital length of stay (OR (95%CI) = 1.87 (0.87–4.02), *p* = 0.1). Finally, using a pre-hospital lactatemia threshold of 4 mmol/L for dichotomization, both subgroups were significantly associated with 30-day mortality: (OR (95%CI) = 3.57 (1.56–9.30), *p* = 0.004) significant for pre-hospital lactatemia ≥ 4 mmol/L and also significant for pre-hospital lactatemia < 4 mmol/L: OR (95%CI) = 0.28 (0.10–0.64), *p* = 0.04).

### 3.3. Propensity Score Matching Analysis

The comparison of variables included in the propensity score showed no significant difference between both subgroups whatever the matching considered (Table 4).

**Figure 2:**
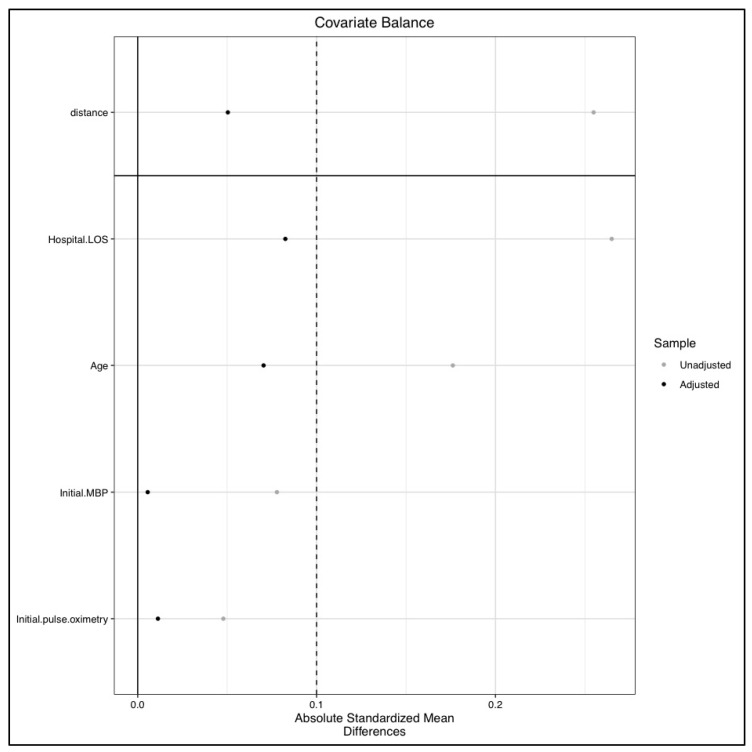
Absolute mean differences between patients with pre-hospital lactatemia ≥ 4 mmol/L and those with pre-hospital lactatemia level < 4 mmol/L after matching. Legend: LOS: length of stay, MBP: mean blood pressure.

Absolute mean differences between subgroups after matching are presented in Figure 2.

After adjustment on confounders, the association with 30-day mortality remains significant: ORa (95%CI) = 2.95 (1.14–9.18), *p* ≤ 10^−3^) for pre-hospital lactatemia ≥ 4 mmol/L.

### 3.4. Survival Analysis 

**Figure 3:**
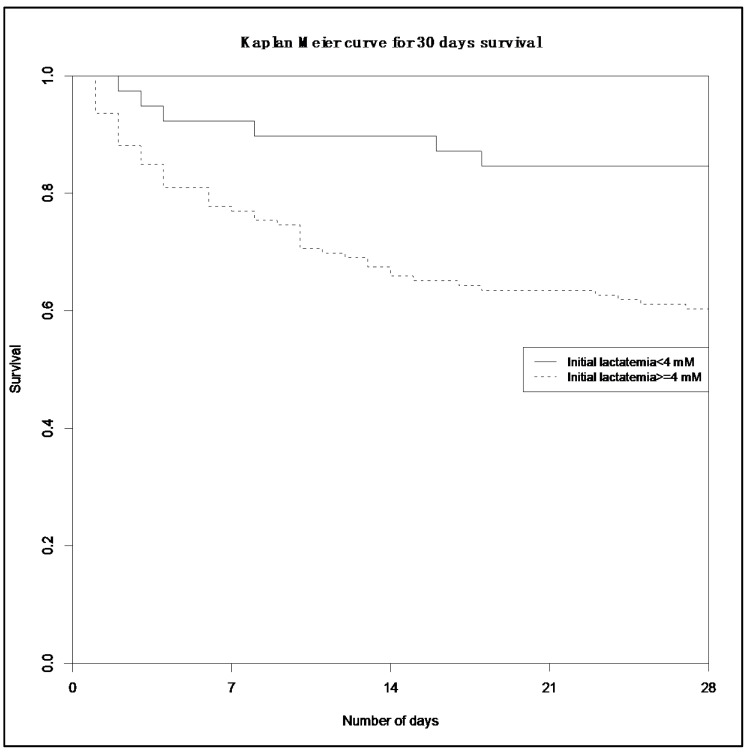
Kaplan Meier curve of 30-day survival.

Using Cox regression analysis, we observed a statistically significant association between pre-hospital lactatemia ≥ 4 mmol/L and 30-day mortality with a hazard ratio (HR) of 2.27 (95%CI (1.02–5.05), *p* = 0.04). Differences in 30-day survival after confounder adjustment in both subgroups was represented by Kaplan–Meier curves (Figure 3).

Using Cox regression analysis adjusted for confounders, we observed an adjusted HR of 30-day mortality for pre-hospital lactatemia ≥ 4 mmol/L (HRa = 2.37, 95%CI (1.01–5.57), *p* = 0.04).

## 4. Discussion

In this study, we observed that pre-hospital lactatemia predicted 30-day mortality for patients with septic shock requiring a MICU. Our results were consistent with previous in-hospital findings [14,15,16,17] which had firstly highlighted the relationship between lactatemia and mortality and secondly that a level of blood lactate higher than 4 mmol/L predicted mortality [12]. Early medical assessment of patients with SS is crucial in triage decision-making process, in instauration of treatment and in optimal orientation of patients between the ICU and the ED. Since 2016, the “sepsis 3” conference and the Center for Disease Control and Prevention ruled that early recognition, severity assessment and treatment are priorities to improve the survival rate of sepsis [8]. Briefly, patients without severity criteria are referred to hospital wards, whereas sicker patients or those at risk of deterioration required an ICU level of care. Evaluation of severity in patients with sepsis is based first on medical history and clinical signs [40,41,42] and second on biomarkers. ICU prognostic scores such as SOFA [21], IGS II and SAPS I [22], combining clinical signs and biological markers, were developed to improve the sensitivity and specificity for the diagnosis of sepsis and the evaluation of its severity. Unfortunately, these scores were not directly transposable to a pre-hospital emergency setting because they are time consuming and require biological data obtained with central laboratory analyzers (24 h for SAPS II and IGS II). In order to avoid this limitation, the 2016 “sepsis 3” conference defined a sepsis severity assessment tool named qSOFA score [8], usable both in the ED and in the pre-hospital setting. Nevertheless, its validity remains a matter of debate because conflicting results obtained with this score were observed between pre-hospital and ED studies [43,44,45,46,47,48,49]. Thus, the use of biomarkers in a pre-hospital setting obtained with mobile devices seems promising to improve the diagnosis of sepsis and the evaluation of its severity. Interestingly, blood lactate was approved as an in-hospital prognostic biomarker in the severity assessment of sepsis [14,15,16]. Moreover, a previous study reported the reliability and transferability of blood lactate measurements between central laboratory analyzers and point-of-care testing [18]. Using a mobile device to assess blood lactate level in a pre-hospital setting, we showed that lactatemia is a strong predictor of worse clinical outcomes in patients with SS. Indeed, we observed that a threshold of 4 mmol/L allowed us to distinguish patients with favorable or unfavorable outcome. Further studies will be needed to identify the optimal pre-hospital triage tool for patients with sepsis: lactatemia alone or in combination with clinical scores (qSOFA, MRST, MEWS and PRESEP). Such a tool will result in a more rapid identification of patients with severe sepsis allowing their early in-ICU admission in the hope of reducing mortality by saving valuable minutes during the early “golden hours”. Finally, this study presents some limitations that should be considered. First, our results may not be transferred to pediatric patients because they were obtained from adult patients. Second, our results cannot be extrapolated to sepsis since those patients were excluded from the analysis according to the study design. Third, our study was conducted in France where the pre-hospital system based on SAMU allows early triage and medical treatment administration in a pre-hospital setting. Fourth, beyond the prognostic value of pre-hospital lactatemia, it would be more informative to measure lactate clearance during the pre-hospital setting. Fifth, we cannot rule out the potential role of other lactate sources affecting the overall lactate concentration such as oral antidiabetic drugs and/or intoxication by a lactate giver (ethanol).

## 5. Conclusions

Pre-hospital lactatemia predicted 30-day mortality for patients with SS requiring a MICU. The value of initial pre-hospital blood lactatemia assessments alone or in association with clinical scores in triage decision-making processes of those patients needs to be clarified. Further studies will be needed to evaluate the contribution of pre-hospital lactatemia to reduce mortality by optimizing pre-hospital triage for patients with SS.

## Figures and Tables

**Table 1:** Population characteristics.

	Overall Population (*n* = 177)	Living (*n* = 118)	Deceased (*n* = 59)	*p* Value
Age (years)	70 ± 14	68 ± 14	74 ± 13	0.009 *
Weight (kg)	70 ± 15	71 ± 15	69 ± 14	0.346
Size (cm)	171 ± 8	171 ± 8	170 ± 9	0.827
SBP (mmHg)	101 ± 55	103 ± 65	97 ± 26	0.483
DBP (mmHg)	58 ± 22	60 ± 23	56 ± 20	0.345
MBP (mmHg)	71 ± 23	72 ± 24	69 ± 20	0.486
HR (beats.min^−1^)	116 ± 29	117 ± 29	113 ± 31	0.386
RR (movements.min^−1^)	30 (24–40)	30 (22–40)	32 (25–38)	0.676
Pulse oximetry (%)	92 (84–96)	91 (85–96)	92 (83–95)	0.568
Body core temperature (°C)	38.3 (36.0–39.2)	38.4 (36.7–39.4)	38.2 (35.6–39.0)	0.521
Glycemia (mmol/L)	8.8 (6.3–12.1)	9.1 (6.9–12.3)	7.3 (5.4–9.7)	0.031 *
Glasgow coma scale	14 (12–15)	14 (12–15)	14 (11–15)	0.560
Blood lactate level (mmol/L)	6.3 ± 3.7	5.9 ± 3.5	7.1 ± 4.0	<0.001 *
Pre-hospital fluid expansion (mL)	1039 ± 587	1067 ± 592	980 ± 575	0.360
Norepinephrine administration	64 (36%)	44 (37%)	20 (34%)	0.838
Norepinephrine dose	1.0 (0.5–2.0)	1.0 (0.5–2.0)	1.0 (0.7–2.0)	0.670
Pre-hospital duration (min)	83 ± 29	83 ± 29	83 ± 29	0.970
In-ICU length of stay (days)	6 (3–10)	7 (4–10)	6 (2–10)	0.146
In-hospital length of stay (days)	14 (8–22)	17 (10–29)	7 (2–13)	<0.001 *
SOFA score	8 (4–11)	6 (3–10)	10 (8–12)	<0.001 *
SAPS2 score	58 ± 22	53 ± 19	70 ± 25	<0.001 *
Male gender	124 (70%)	85 (72%)	39 (66%)	0.743
High blood pressure	78 (44%)	52 (44%)	26 (44%)	0.775
Coronaropathy	25 (22%)	15 (13%)	10 (17%)	0.981
Chronic cardiac failure	21 (12%)	11 (9%)	10 (17%)	0.113
Diabetes mellitus	46 (26%)	36 (31%)	10 (17%)	0.081
HIV infection	6 (3%)	4 (3%)	2 (3%)	0.952
Cancer history	53 (30%)	36 (31%)	17 (29%)	0.981
COPD	17 (10%)	11 (9%)	6 (10%)	0.774
Chronic renal failure	19 (11%)	11 (9%)	8 (14%)	0.332
Pre-hospital AB administration	54 (31%)	37 (31%)	17 (29%)	0.890

Results were expressed as mean and standard deviation for quantitative parameters (normal distribution), as median and interquartile range for quantitative parameters (non-gaussian distribution) and, as absolute value and percentage for qualitative parameters. *p*-value corresponds to the comparison between deceased and living patients. SBP = systolic blood pressure, DBP = diastolic blood pressure, MBP = mean blood pressure, HR = heart rate, RR = respiratory rate, ICU = intensive care unit, SOFA = sequential organ failure assessment, SAPS2 = simplified acute physiology score 2nd version, HIV = human immunodeficiency virus, COPD = chronic obstructive pulmonary disease, AB = antibiotic, min = minutes. * *p*-value < 0.05 between living and deceased patients on day-30.

**Table 2:** Presumed septic shock origins.

Origin	*n* (percentage)
Pulmonary	102 (58%)
Digestive	38 (21%)
Urinary	19 (11%)
Cutaneous	5 (3%)
Meningeal	3 (2%)
Unknown	10 (6%)

**Table 3:** Comparison between patients with initial pre-hospital lactatemia <4 mmol/L and those with initial pre-hospital lactatemia ≥4 mmol/L.

	Overall Population (*n* = 177)	Pre-hospital Lactatemia ≥ 4 mmol/L (*n* = 57)	Pre-hospital Lactatemia < 4 mmol/L (*n* = 120)	*p* Value
Age (years)	70 ± 14	69 ± 15	71 ± 12	0.483
Weight (kg)	70 ± 15	72 ± 14	69 ± 16	0.300
Size (cm)	171 ± 8	171 ± 8	170 ± 10	0.663
SBP (mmHg)	101 ± 55	99 ± 29	91 ± 31	0.367
DBP (mmHg)	58 ± 22	59 ± 21	57 ± 22	0.612
MBP (mmHg)	71 ± 23	70 ± 22	69 ± 24	0.793
HR (beats.min^−1^)	116 ± 29	118 ± 29	109 ± 29	0.069
RR (movements.min^−1^)	30 (24–40)	32 (25–40)	28 (20–36)	0.038 *
Pulse oximetry (%)	92 (84–96)	92 (83–97)	90 (85–95)	0.824
Body core temperature (°C)	38.3 (36.0–39.2)	38.0 (35.6–39.1)	38.6 (38.0–39.2)	0.022 *
Glycemia (mmol/L)	8.8 (6.3–12.1)	9.0 (6.4–12.3)	7.5 (6.0–10.2)	0.281
Glasgow coma scale	14 (12–15)	14 (12–15)	15 (12–15)	0.594
Blood lactate level (mmol/L)	6.3 ± 3.7	2.3 ± 1.0	7.7 ± 3.2	<0.001 *
Pre-hospital fluid expansion (mL)	1039 ± 587	1038 ± 599	979 ± 547	0.551
Norepinephrine administration	64 (36%)	18 (32%)	46 (38%)	0.921
Norepinephrine dose	1.0 (0.5–2.0)	1.0 (0.5–2.0)	1.0 (0.9–1.1)	0.185
Pre-hospital duration (min)	83 ± 29	81 ± 30	85 ± 28	0.486
In-ICU length of stay (days)	6 (3–10)	7 (4–11)	5 (3–8)	0.083
In-hospital length of stay (days)	14 (8–22)	14 (7–23)	15 (8–20)	0.351
SOFA score	8 (4–11)	8 (5–11)	3 (5–8)	0.026 *
SAPS2 score	58 ± 22	63 ± 22	49 ± 21	<0.001 *
Male gender	124 (70%)	31 (54%)	93 (78%)	0.401
High blood pressure	78 (44%)	17 (30%)	61 (51%)	0.494
Coronaropathy	25 (22%)	3 (5%)	22 (18%)	0.093
Chronic cardiac failure	21 (12%)	9 (16%)	12 (10%)	0.272
Diabetes mellitus	46 (26%)	11 (19%)	35 (30%)	0.675
HIV infection	6 (3%)	1 (2%)	5 (3%)	0.473
Cancer history	53 (30%)	15 (26%)	38 (32%)	0.143
COPD	17 (10%)	4 (7%)	13 (11%)	0.788
Chronic renal failure	19 (11%)	3 (5%)	16 (13%)	0.786
Pre-hospital AB administration	54 (31%)	20 (35%)	34 (28%)	0.177

Results were expressed as mean and standard deviation for quantitative parameters (normal distribution), as median and interquartile range for quantitative parameters (non-gaussian distribution) and, as absolute value and percentage for qualitative parameters. *p*-value corresponds to the comparison between initial pre-hospital lactatemia <4 mmol/L and initial pre-hospital lactatemia ≥4 mmol/L. Legend: SBP = systolic blood pressure, DBP = diastolic blood pressure, MBP = mean blood pressure, HR = heart rate, RR = respiratory rate, ICU = intensive care unit, SOFA = sequential organ failure assessment, SAPS2 = simplified acute physiology score 2nd version, HIV = human immunodeficiency virus, COPD = chronic obstructive pulmonary disease, AB = antibiotic, min = minutes. * *p*-value < 0.05 between initial pre-hospital lactatemia <4 mmol/L and initial pre-hospital lactatemia ≥ 4 mmol/L.

**Table 4:** Comparison of variables for 30-day mortality included in the propensity score before and after matching.

	Before Matching *n* = 171			After Matching *n* = 166		
PS covariate	IPL < 4 mM *n* = 126	IPL ≥ 4 mM *n* = 45	*p* Value	IPL < 4 mM *n* = 126	IPL ≥ 4 mM *n* = 40	*p* Value
Age	71 ± 12	69 ± 15	0.395	71 ± 12	69 ± 15	0.481
In-hospital LOS	15 (8–20)	14 (7–23)	0.355	15 (8–20)	14 (7–23)	0.328
Initial PO	90 (85–94)	92 (83–97)	0.777	90 (85–94)	92 (83–97)	0.717
Initial MBP	69 ± 24	71 ± 22	0.631	69 ± 20	71 ± 22	0.657

Values are expressed as mean ± SD or number (%). PS: propensity score, LOS: length of stay, initial PO: initial pulse oximetry, initial MBP: initial mean blood pressure, IPL: initial pre-hospital lactatemia, mM = mmol/L.
